# Dephosphorylated-uncarboxylated Matrix Gla protein concentration is predictive of vitamin K status and is correlated with vascular calcification in a cohort of hemodialysis patients

**DOI:** 10.1186/1471-2369-15-145

**Published:** 2014-09-04

**Authors:** Pierre Delanaye, Jean-Marie Krzesinski, Xavier Warling, Martial Moonen, Nicole Smelten, Laurent Médart, Hans Pottel, Etienne Cavalier

**Affiliations:** Nephrology-Dialysis-Transplantation, University of Liège, CHU Sart Tilman, Liège, Belgium; Nephrology-Dialysis, Centre Hospitalier Régional “La Citadelle”, Liège, Belgium; Nephrology-Dialysis, Centre Hospitalier “Bois de l’Abbaye”, Seraing, Belgium; Radiology, Centre Hospitalier “La Citadelle”, Liège, Belgium; Interdisciplinary Research Center, University of Leuven, Kulak, Kortrijk, Belgium; Clinical Chemistry, University of Liège, CHU Sart Tilman, Liège, Belgium

**Keywords:** Matrix Gla protein, Vascular calcification, Vitamin K

## Abstract

**Background:**

Matrix Gla protein (MGP) is known to act as a potent local inhibitor of vascular calcifications. However, in order to be active, MGP must be phosphorylated and carboxylated, with this last process being dependent on vitamin K. The present study focused on the inactive form of MGP (dephosphorylated and uncarboxylated: dp-ucMGP) in a population of hemodialyzed (HD) patients. Results found in subjects being treated or not with vitamin K antagonist (VKA) were compared and the relationship between dp-ucMGP levels and the vascular calcification score were assessed.

**Methods:**

One hundred sixty prevalent HD patients were enrolled into this observational cohort study, including 23 who were receiving VKA treatment. The calcification score was determined (using the Kauppila method) and dp-ucMGP levels were measured using the automated iSYS method.

**Results:**

dp-ucMGP levels were much higher in patients being treated with VKA and little overlap was found with those not being treated (5604 [3758; 7836] vs. 1939 [1419; 2841] pmol/L, p <0.0001). In multivariate analysis, treatment with VKA was the most important variable explaining variation in dp-ucMGP levels even when adjusting for all other significant variables. In the 137 untreated patients, dp-ucMGP levels were significantly (p < 0.05) associated both in the uni- and multivariate analysis with age, body mass index, plasma levels of albumin, C-reactive protein, and FGF-23, and the vascular calcification score.

**Conclusion:**

We confirmed that the concentration of dp-ucMGP was higher in HD patients being treated with VKA. We observed a significant correlation between dp-ucMGP concentration and the calcification score. Our data support the theoretical role of MGP in the development of vascular calcifications. We confirmed the potential role of the inactive form of MGP in assessing the vitamin K status of the HD patients.

**Trial registration:**

B707201215885

## Background

Matrix Gla protein (MGP) is an 11 kDa protein secreted by chondrocytes and vascular smooth muscle cells (VSMCs) [1]. MGP acts as a potent local inhibitor of vascular calcifications by directly inhibiting calcium precipitation and crystallization [2, 3] and/or by antagonizing bone morphogenetic protein (BMP2), which itself promotes osteoblastic differentiation of VSMCs [4]. MGP-deficient mice have been shown to develop excessive and premature arterial calcifications, leading to death by rupture of the aorta in the first two months of life [2]. In order to be fully active, MGP must first undergo two posttranslational processes: the phosphorylation of three serine residues (although the role of this phosphorylation process is still not well understood) and the carboxylation of five glutamate residues [5, 6]. This explains why it is theoretically possible for several different isoforms of MGP to be measured in the plasma (a combination of carboxylated, uncarboxylated and phosphorylated, unphosphorylated MGP). Different fragments of MGP may, in fact, have different physiological roles and meanings [7, 8]. In this article, we will focus on the inactive form of MGP (dephosphorylated and uncarboxylated: dp-ucMGP). Because γ-glutamyl carboxylation is highly dependent on availability of vitamin K [9], it is possible that the measurement of dp-ucMGP would reflect vitamin K status [7, 10].

In hemodialysis (HD) patients, vascular calcifications are precocious, frequent and excessive [11–14]. The association between the level of vascular calcifications and mortality has been described in HD patients in various studies [15–17]. There are several *in vitro* and *in vivo* data suggesting a direct link between the decreased availability of vitamin K and vascular calcification, based on the role of this vitamin in the activation of MGP [9, 18]. Various authors have described a decreased availability of vitamin K (both K_1_ and K_2)_ in patients with chronic kidney disease (CKD) [19–24]. As a result, the level of the inactive form, dp-ucMGP, has been found to increase in these patients, in comparison with non-CKD patients [7, 10, 21, 22, 25]. In addition, vitamin K therapy has been shown to significantly decrease the levels of dp-ucMGP both in the general population [8, 26] and HD patients [7, 10, 27]. Conversely, it has been shown in the general population and in CKD patients that vitamin K antagonist (VKA) is associated with higher dp-ucMGP levels [8, 23]. If the same higher concentration is also observed in dialysis patients is not known. These results suggest that dp-ucMGP could reflect a person’s vitamin K status at the vascular level [7, 10, 21, 27–29]. Moreover, it is interesting that, in CKD patients, some authors have found a significant correlation between dp-ucMGP levels and vascular calcifications [25] but this finding has not been confirmed by others [7]. This point is thus still debatable. In this study, we measured dp-ucMGP levels in a cohort of HD patients and compared the results between those being treated or not with VKA. We also assessed the potential relationship between dp-ucMGP levels and the vascular calcification score.

## Methods

Prevalent hemodialysis patients from three independent centers in Liège and the surrounding areas in Belgium, were included in this observational cohort study (Centre Hospitalier Universitaire du Sart Tilman, Centre Hospitalier Regional de La Citadelle, Centre Hospitalier Bois de l’Abbaye de Seraing). From the initial sample (n = 212), we restricted the analysis to patients who were able and agreed to have their vascular calcification score measured (n = 165). Vascular calcifications were assessed by lateral X-ray radiography (the "Kauppila" method) and the score (between 0 to 24) was established by the same experienced investigator (LM) [30, 31]. Of the 165 patients, MGP was not measured in 5 patients, due to technical issues. In the final sample, 23 patients were being treated with VKA (acenocoumarol, Novartis Pharma) and 137 patients were not being treated with this therapy. The following clinical data were considered: age, gender, body mass index (BMI), dialysis vintage, previous cardiovascular disease, hypertension, diabetes and smoking habit. Hypertension was defined as having a blood pressure greater than 140/90 mm Hg and/or being in receipt of treatment for hypertension. Diabetes status was obtained from electronic medical files and/or defined according to being in receipt of treatment for diabetes. Previous cardiovascular disease was defined as having a history of myocardial infarction, percutaneous coronary artery intervention, cardiac surgery, peripheral artery disease or cerebrovascular disease. Data were extracted from electronic medical files and completed through interviews with the patients. Patients were defined as having a smoking habit if they currently smoked. All data from the electronic files have been then confirmed by nephrologists taking care of the patients. The following laboratory data were studied (one-point measurements): plasma levels of calcium, phosphorus, albumin, C-reactive protein (CRP) (measured using the Modular P autoanalyzer, Roche, Mannheim, Germany), intact parathormone (measured using the Elecsys analyzer, Roche, Mannheim, Germany), 25-OH vitamin D, bone-specific alkaline phosphatase (measured using the Liaison analyzer, Diasorin, Stillwater, MN) and C-terminal Fibroblast Growth Factor (FGF-23) (measured by ELISA, Biovendor, Czech Republic). dp-ucMGP was quantified by the first automated method available on the market (supplied by IDS, Boldon, UK). A precision profile was determined using 4 plasma samples (264-2703 pmol/L), measured twice a day for 10 days. CV intra-assays and inter-assays were thus measured at 2.9-8.9% and 4.1-13.4%, respectively. The IDS kit was found to be linear up to a value of 9724 pmol/L. We also measured dp-ucMGP levels using this kit in a healthy population (n = 62) and the expected (normal) range was found to be < 572 pmol/L. Concomitant therapies were available but not included in the final statistical analysis: 63% were treated by low dosage of calcium (carbonate or acetate), 25% by sevelamer, 5% by lanthanum, 66% by cholecalciferol and 29% by active vitamin D.

Human subjects procedures in the present study were in accordance with the ethical standards of the Helsinki Declaration of 1975. All participants provided their written informed consent. The study and the approved consent were approved by the Ethics Committee of the University Hospital, CHU Sart Tilman "Comité d’éthique Hospitalo-Facultaire Universitaire de Liège, http://www.chu.ulg.ac.be/jcms/c_11204/comite-d-ethique-hospitalo-facultaire". The Ethics committee of the University Hospital has the authority to approve the study for all participating sites. The Belgian number of this study is B707201215885. Immunodiagnostic Systems (IDS) PLC provided the kits for measuring dp-ucMGP levels but the company was not involved in the study design, data analysis, or preparation of the manuscript.

### Statistical analysis

Data were expressed as mean ± standard deviation (SD) when the distribution was normal and as being in the median and interquartile range [IQR] if not. As appropriate, the baseline characteristics were compared between treated and non-treated groups using the Student’s t-test, the Mann–Whitney U test or the Chi-square test.

Regression analysis was used to study the potential linear relationship between the vascular calcification score and dp-ucMGP levels. A multivariate analysis was also performed with variables that were found to be associated with dp-ucMGP in the univariate analysis (p = 0.1). We defined tertiles according to dp-ucMGP concentrations and compared the vascular calcification score between the tertiles using the Mann–Whitney and Kruskal-Wallis tests. All statistical analyses were conducted using the Medcalc (Mariakerke, Belgium) and SAS 9.3 (SAS Institute Inc. Cary, NC) software.

## Results

**Figure 1:**
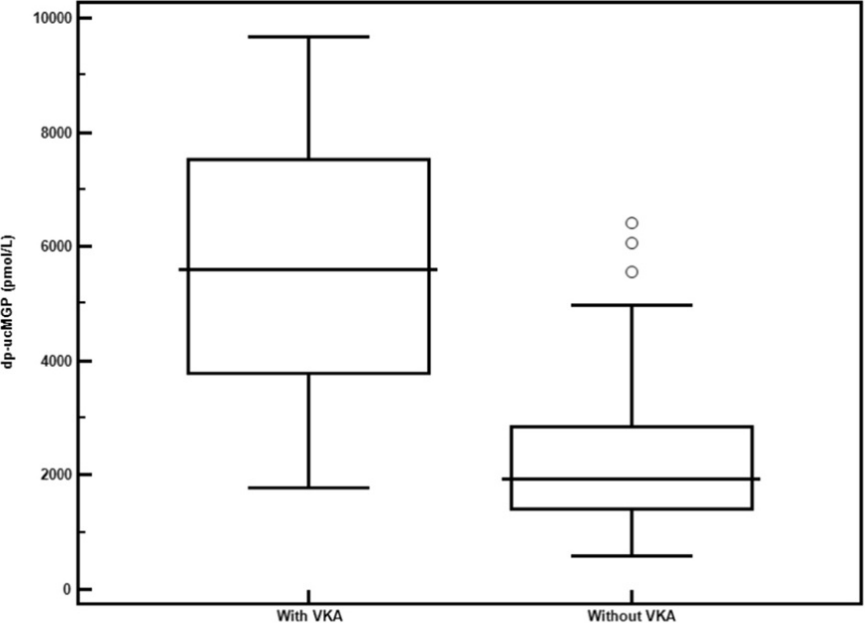
**Median concentration of dp-ucMGP in patients treated with antivitamin K (VKA) (n = 23) and in patients not treated with VKA (n = 137) (5604 [3758; 7836] vs. 1939 [1419; 2841] pmol/L, p <0.0001).**

The clinical and biological variables of the total population of the study (n = 160) are summarized in Table 1. The concentration of dp-ucMGP was high in our HD patients: 2148 [1542; 3251] pmol/L. Table 1 also shows the differences in the clinical and biological variables between the 23 patients being treated with VKA and the 137 not being treated with VKA. No significant difference was observed regarding clinical characteristics, except in the percentage of patients with hypertension, which was slightly higher in patients being treated with VKA. We observed a significant difference in dp-ucMGP levels, with a much higher concentration in patients being treated with VKA and little overlap with patients not being treated with VKA (5604 [3758; 7836] vs. 1939 [1419; 2841] pmol/L, p <0.0001) (Figure 1). In the multivariate analysis (generalized linear model) on all patients, treatment with VKA was, by far, the most important variable explaining variation in dp-ucMGP levels (R^2^ = 0.59, p < 0.0001).

**Table 1:** **Main clinical characteristics and biological data of the total population of the study and according to antivitamin K** (**VKA**) **therapy status**

	Total population	Non-treated with VKA	Treated with VKA	***P*** (between treated and non-treated)
n	160	137	23	
Age (yr)	74 [63; 80]	74 [64; 81]	71 [59; 79]	NS
Male gender (%)	44	43	52	NS
Body mass index (kg/m^2^)	25.4 [22.6; 30.2]	25.4 [22.6; 30.2]	27.3 [24.7; 33.3]	NS
Dialysis vintage (month)	23 [11; 44]	22 [11; 48]	24 [13; 38]	NS
Previous CVD (%)	65	64	74	NS
Hypertension (%)	87	85	100	0.046
Diabetes (%)	44	43	52	NS
Smoking habit (%)	21	21	22	NS
Calcium (mmol/L)	2.15 ± 0.16	2.16 ± 0.15	2.14 ± 0.20	NS
Phosphate (mg/dL)	4.6 [4.0; 6.0]	4.6 [4; 6]	5.1 [4.3; 5.7]	NS
Albumin (g/L)	38 [36; 40]	38 [36; 40]	40 [38; 40]	NS
CRP (mg/L)	5 [2; 13]	5 [2; 13]	4 [3; 9]	NS
Intact PTH (pg/mL)	263 [126; 434]	251 [129; 423]	291 [125; 570]	NS
25-OH vitamin D (ng/mL)	22 [12; 33]	22 [12; 33]	19 [13; 25]	NS
b-ALP (μg/L)	16 [11; 23]	15 [10; 23]	19 [13; 29]	NS
FGF-23 (RU/mL)	2911 [1039; 7413]	2733 [883; 7597]	3179 [2391; 6676]	NS
Calcification score (maximum score is 24)	10 [5; 15]	10 [5; 15]	11 [6; 17]	NS
dp-ucMGP (pmol/L)	2148 [1542; 3251]	1939 [1419; 2841]	5604 [3758; 7836]	<0.0001

Note: conversion factor for units: phosphorus in mg/dL to mmol/L, x0.3229. Data are expressed as mean ± standard deviation (SD) when distribution was normal and as median and interquartile range [percentile 25; percentile 75] when not. CVD, cardiovascular disease; CRP, C-reactive protein; PTH, parathormone; b-ALP bone-specific alkaline phosphatase; dp-ucMGP, dephosphorylated and uncarboxylated; NS, non significant (p > 0.05).

**Figure 2:**
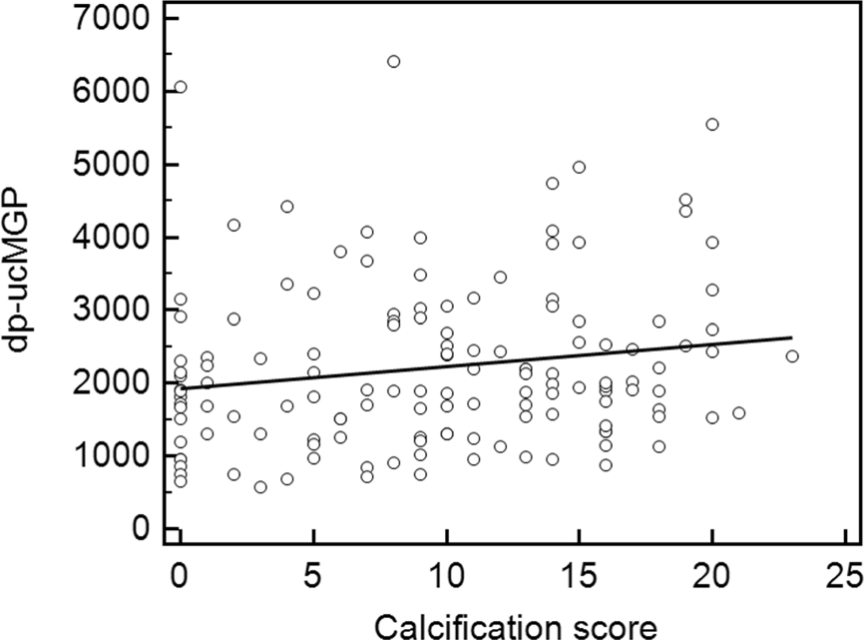
**Univariate regression between the calcification score and dp-ucMGP (in pmol/L) in patients not treated with VKA (n = 137) (r** ^**2**^ **= 0.02850, p = 0.049).**

In the 137 patients not being treated with VKA, dp-ucMGP levels were significantly (p < 0.05) associated in the univariate analysis with age and BMI, and with plasma levels of albumin, CRP and FGF-23. All these associations were positive, except with albumin. Also, a slight but significant correlation was found between dp-ucMGP levels and the calcification score (r = 0.17, p = 0.049) (Figure 2). In the multivariate model, dp-ucMGP levels were significantly associated with BMI (p = 0.0032), and with plasma levels of albumin (p = 0.0368), FGF-23 (p = 0.002) and CRP (p = 0.0012), as well as with the calcification score (p = 0.0206) (Table 2).

**Table 2:** **Variables associated with dp**-**ucMGP concentrations in the multivariate model**

	r	p
Body mass index	0.17	0.0032
Albumin	-0.24	0.0368
FGF-23	0.28	0.002
CRP	0.33	0.0012
Calcification score	0.19	0.0206

Note: r is the zero order correlation coefficient for the variable in the univariate analysis. p is the p value of the variable in the multivariate analysis. CRP, C-reactive protein; FGF, Fibroblast Growth Factor.

**Figure 3:**
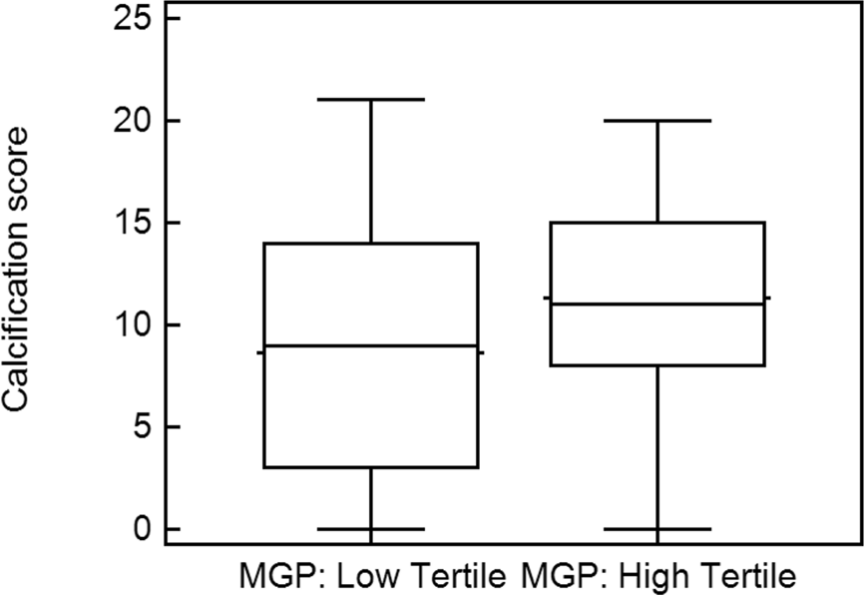
**Comparison of the calcification score in the highest** ***versus*** **the lowest tertile of dp-ucMGP (in pmol/L) (9 ± 6** ***versus*** **11 ± 6, p = 0.0414) in patients not treated with VKA (n = 137).**

Considering the tertiles of dp-ucMGP levels (at 1647 and 2404 pmol/L), subjects in the highest tertile (tertile 3) had a significantly higher vascular calcification score than those in tertile 1 (but not compared with tertile 2): 9 ± 6 *versus* 11 ± 6 (p = 0.0414) (Figure 3). Compared to tertile 1, tertile 3 included more men with a higher BMI and with lower albumin levels.

## Discussion

Our results confirmed that dp-ucMGP concentrations are higher in dialysis patients with a median concentration of 2148 [1542; 3251] pmol/L, whereas the normal range was found to be < 572 pmol/L in 62 healthy subjects. We also showed for the first time that dialysis patients treated by VKA have significantly higher dp-ucMGP concentrations than not treated dialysis patients. Lastly, we showed a mild but significant correlation between dp-ucMGP and vascular calcifications. We confirmed that the concentration of dp-ucMGP was higher in dialysis patients than in the healthy population we had also tested. Indeed, the median concentration in our HD population was: 2148 [1544; 3237] pmol/L, which is much higher than concentrations observed elsewhere in non-CKD and CKD non-HD populations [7, 23, 25, 26, 32]. The mean concentration of dp-ucMGP in our population (2704 ± 1798 pmol/L) is however very comparable to the concentration observed by Schlieper *et al*. in their 188 HD subjects (2850 ± 1768 pmol/L) [7]. This high concentration of dp-ucMGP in HD patients is not fully understood. These patients are clearly sensitive to vitamin K deficiency, and this is likely because the recommended diet in HD patients is deficient in vitamin K [19–22, 24]. However, decreased excretion or catabolism of MGP associated with decreased glomerular filtration is also a plausible explanation for the molecular weight of MGP (11 kDa) [7, 33]. In addition, a classical exponential relationship has been observed between dp-ucMGP and estimated glomerular filtration rate [25]. Lastly, the burden of vascular calcifications in HD patients could also influence the level of dp-ucMGP (see below) [25, 32].

For the first time in HD patients, we showed here that VKA treatment strongly and independently impacted, and actually increased the levels of dp-ucMGP, although the clinical and biological profiles of these patients were very comparable to HD patients not receiving this therapy. Vitamin K is necessary for the carboxylation of MGP and is thus a key vitamin in the process of MGP activation [9, 18]. An increase in the inactive form of the protein, dp-ucMGP is thus to be expected. Such an increase in dp-ucMGP levels in VKA treated patients has already been shown in the general and in CKD populations but not in HD populations [8, 23, 25]. In HD patients, some authors have demonstrated a decrease in dp-ucMGP levels associated with vitamin K therapy [7, 10, 27]. Our results confirm that patients being treated with VKA must be considered separately in studies involving MGP and underline the potential role of dp-ucMGP in monitoring the vitamin K status of HD patients [34].

Because MGP is a potent inhibitor of vascular calcifications [1, 2, 4], some authors have suggested that the protein may be useful in the monitoring or even the detection of vascular calcifications in CKD or HD subjects [25, 35]. In the present study, we confirmed a significant correlation between dp-ucMGP levels and the calcification score in the univariate analysis and this association was confirmed in the multivariate model. Similarly, we found that the patients in the highest tertile of dp-ucMGP levels had a significantly higher calcification score than the patients in the lowest tertile. Our results confirm those published by Schurgers *et al*., who also showed a positive association (both in univariate and multivariate analysis) between dp-ucMGP levels and the calcification score in 107 CKD patients (including 40 HD patients) [25]. Schlieper *et al*, however, did not confirm any relationship between dp-ucMGP levels and the calcification score in 188 HD patients [7]. Interestingly, these two authors used an ELISA method to measure dp-ucMGP, which could explain, at least in part, some discrepancies between their studies [7, 8, 25]. For the first time, we used an automated assay with a high analytical performance. Another potential explanation of discrepant results could be the differences in techniques applied for calcification detection. Schurgers and colleagues used the most sensitive technique, i.e. multislice spiral computed tomography [25], Schlieper used a modified Adragao score [15], which is an extension of the Kauppila method [7] and we used the classical Kauppila method, as recommended by the KDIGO guidelines [30]. In our prevalent HD population, the correlation between dp-ucMGP levels and the calcification score remained relatively poor. Also, the overlap between the MGP tertiles regarding the calcification score was high, making the measurement of dp-ucMGP levels uncertain, at the individual level, in detecting or estimating the burden of the calcification score. This positive correlation between dp-ucMGP levels and the calcification score supports however the theoretical role of MGP in the development of vascular calcifications. It also advocates the need for additional studies, particularly with other active isoforms of MGP [7, 22, 32, 35–39]. For instance, total uncarboxylated MGP (which may be partially bioactive) has been negatively associated with coronary calcification in dialysis patients [36]. Moreover, reduced levels of non-phosphorylated carboxylated MGP (also partially bioactive form of MGP) has been associated with increased cardiovascular mortality and with vascular calcifications in dialysis patients [7]. An human ELISA for the measurement of the fully bioactive form (phosphorylated, carboxylated MGP) is still not available on the market. The positive association described in the present study between dp-ucMGP and BMI or CRP has also been described by others [25, 26]. The negative association discovered here with plasma albumin and the positive one with FGF-23 levels, and with dialysis vintage, on the other hand, has not been found to date in HD patients, and this deserves further investigations.

There are several limitations to our study. First, our population was a prevalent HD population and the study is observational. Further studies with incident cases and follow-up of hard endpoints would be welcome. Moreover, our HD population was relatively older (71 ± 17 years old), in comparison with other studies (67 ± 13 y in Schurgers’s study [25] and 59 ± 11 y in Schlieper [7]). Because age is a well-known determinant of vascular calcifications, different results might be observed in younger HD patients. Second, it would be interesting to confirm whether patients being treated with VKA have a higher risk of vascular calcifications. This was not the case in our population (data not shown) but our sample of prevalent VKA treated patients (n = 23) was probably too small. Several authors have suggested that VKA treatment is a risk factor for vascular calcifications or calciphylaxis in HD patients but this assertion deserves further study [24, 32, 35, 39–46]. Third, the Kauppila method is not the most sensitive way of detecting vascular calcifications and we did not directly measure neither vitamin K levels nor other vitamin K dependent protein like PIVKA-II (protein induced by vitamin K absence II). However, the Kauppila method is the approach recommended by the KDIGO [30]. Lastly, we do not have precise data regarding the VKA adequacy (INR are not available) and the duration of VKA therapy in all patients. However, VKA is widely prescribed for atrial fibrillation or valve replacement and therefore, long term therapy must be considered in the majority of our treated patients.

## Conclusions

In this observational study, we confirmed high levels of dp-ucMGP in HD patients. For the first time, we used an automated and efficient assay to measure dp-ucMGP. We confirmed the potential usefulness of MGP (dp-ucMGP or another active isoform) in the assessment of vascular calcifications, although this finding has, to date, been the subject of debate in the literature. For the first time, we showed that dp-ucMGP levels were much higher in HD patients being treated with VKA. This observation underlines the specific role of the inactive form of MGP, dp-ucMGP (measured by the automated assay) in assessing vitamin K status. This measurement could be of great interest in monitoring the vitamin K therapy if future randomized studies, notably the VitaVasK trial [34], confirm the effectiveness of this vitamin in HD patients [18, 28, 32, 34, 35, 39, 44, 47, 48].

## Abbreviations

b-ALP: bone-specific alkaline phosphatase
BMI: body mass index
CKD: Chronic kidney disease
dp-ucMGP: dephosphorylated and uncarboxylated
FGF-23: C-terminal Fibroblast Growth Factor
HD: Hemodialyzed
MGP: Matrix Gla protein
PTH: Parathormone
VKA: vitamin K antagonist
VSMC: Vascular smooth muscle cells.

## References

[1] Price PA, Urist MR, Otawara Y (1983). Matrix Gla protein, a new gamma-carboxyglutamic acid-containing protein which is associated with the organic matrix of bone. Biochem Biophys Res Commun.

[2] Luo G, Ducy P, McKee MD, Pinero GJ, Loyer E, Behringer RR, Karsenty G (1997). Spontaneous calcification of arteries and cartilage in mice lacking matrix GLA protein. Nature.

[3] Lomashvili KA, Wang X, Wallin R, O’Neill WC (2011). Matrix Gla protein metabolism in vascular smooth muscle and role in uremic vascular calcification. J Biol Chem.

[4] Zebboudj AF, Imura M, Bostrom K (2002). Matrix GLA protein, a regulatory protein for bone morphogenetic protein-2. J Biol Chem.

[5] Murshed M, Schinke T, McKee MD, Karsenty G (2004). Extracellular matrix mineralization is regulated locally; different roles of two gla-containing proteins. J Cell Biol.

[6] Schurgers LJ, Spronk HM, Skepper JN, Hackeng TM, Shanahan CM, Vermeer C, Weissberg PL, Proudfoot D (2007). Post-translational modifications regulate matrix Gla protein function: importance for inhibition of vascular smooth muscle cell calcification. J Thromb Haemost.

[7] Schlieper G, Westenfeld R, Kruger T, Cranenburg EC, Magdeleyns EJ, Brandenburg VM, Djuric Z, Damjanovic T, Ketteler M, Vermeer C, Dimkovic N, Floege J, Schurgers LJ (2011). Circulating nonphosphorylated carboxylated matrix gla protein predicts survival in ESRD. J Am Soc Nephrol.

[8] Cranenburg EC, Koos R, Schurgers LJ, Magdeleyns EJ, Schoonbrood TH, Landewe RB, Brandenburg VM, Bekers O, Vermeer C (2010). Characterisation and potential diagnostic value of circulating matrix Gla protein (MGP) species. Thromb Haemost.

[9] Schurgers LJ, Cranenburg EC, Vermeer C (2008). Matrix Gla-protein: the calcification inhibitor in need of vitamin K. Thromb Haemost.

[10] Westenfeld R, Krueger T, Schlieper G, Cranenburg EC, Magdeleyns EJ, Heidenreich S, Holzmann S, Vermeer C, Jahnen-Dechent W, Ketteler M, Floege J, Schurgers LJ (2012). Effect of vitamin K2 supplementation on functional vitamin K deficiency in hemodialysis patients: a randomized trial. Am J Kidney Dis.

[11] Braun J, Oldendorf M, Moshage W, Heidler R, Zeitler E, Luft FC (1996). Electron beam computed tomography in the evaluation of cardiac calcification in chronic dialysis patients. Am J Kidney Dis.

[12] Goodman WG, Goldin J, Kuizon BD, Yoon C, Gales B, Sider D, Wang Y, Chung J, Emerick A, Greaser L, Elashoff RM, Salusky IB (2000). Coronary-artery calcification in young adults with end-stage renal disease who are undergoing dialysis. N Engl J Med.

[13] Jean G, Bresson E, Terrat JC, Vanel T, Hurot JM, Lorriaux C, Mayor B, Chazot C (2009). Peripheral vascular calcification in long-haemodialysis patients: associated factors and survival consequences. Nephrol Dial Transplant.

[14] Oh J, Wunsch R, Turzer M, Bahner M, Raggi P, Querfeld U, Mehls O, Schaefer F (2002). Advanced coronary and carotid arteriopathy in young adults with childhood-onset chronic renal failure. Circulation.

[15] Adragao T, Pires A, Birne R, Curto JD, Lucas C, Goncalves M, Negrao AP (2009). A plain X-ray vascular calcification score is associated with arterial stiffness and mortality in dialysis patients. Nephrol Dial Transplant.

[16] London GM, Guerin AP, Marchais SJ, Metivier F, Pannier B, Adda H (2003). Arterial media calcification in end-stage renal disease: impact on all-cause and cardiovascular mortality. Nephrol Dial Transplant.

[17] Schlieper G, Kruger T, Djuric Z, Damjanovic T, Markovic N, Schurgers LJ, Brandenburg VM, Westenfeld R, Dimkovic S, Ketteler M, Grootendorst DC, Dekker FW, Floege J, Dimkovic N (2008). Vascular access calcification predicts mortality in hemodialysis patients. Kidney Int.

[18] Schurgers LJ (2013). Vitamin K: key vitamin in controlling vascular calcification in chronic kidney disease. Kidney Int.

[19] Holden RM, Morton AR, Garland JS, Pavlov A, Day AG, Booth SL (2010). Vitamins K and D status in stages 3–5 chronic kidney disease. Clin J Am Soc Nephrol.

[20] Pilkey RM, Morton AR, Boffa MB, Noordhof C, Day AG, Su Y, Miller LM, Koschinsky ML, Booth SL (2007). Subclinical vitamin K deficiency in hemodialysis patients. Am J Kidney Dis.

[21] Cranenburg EC, Schurgers LJ, Uiterwijk HH, Beulens JW, Dalmeijer GW, Westerhuis R, Magdeleyns EJ, Herfs M, Vermeer C, Laverman GD (2012). Vitamin K intake and status are low in hemodialysis patients. Kidney Int.

[22] Fusaro M, Noale M, Viola V, Galli F, Tripepi G, Vajente N, Plebani M, Zaninotto M, Guglielmi G, Miotto D, Dalle CL, D'Angelo A, Naso A, Grimaldi C, Miozzo D, Giannini S, Gallieni M (2012). Vitamin K, vertebral fractures, vascular calcifications, and mortality: VItamin K Italian (VIKI) dialysis study. J Bone Miner Res.

[23] Boxma PY, van den Berg E, Geleijnse JM, Laverman GD, Schurgers LJ, Vermeer C, Kema IP, Muskiet FA, Navis G, Bakker SJ, de Borst MH (2012). Vitamin k intake and plasma desphospho-uncarboxylated matrix Gla-protein levels in kidney transplant recipients. PLoS One.

[24] Voong K, Harrington D, Goldsmith D (2013). Vitamin K status in chronic kidney disease: a report of a study and a mini-review. Int Urol Nephrol.

[25] Schurgers LJ, Barreto DV, Barreto FC, Liabeuf S, Renard C, Magdeleyns EJ, Vermeer C, Choukroun G, Massy ZA (2010). The circulating inactive form of matrix gla protein is a surrogate marker for vascular calcification in chronic kidney disease: a preliminary report. Clin J Am Soc Nephrol.

[26] Shea MK, O’Donnell CJ, Vermeer C, Magdeleyns EJ, Crosier MD, Gundberg CM, Ordovas JM, Kritchevsky SB, Booth SL (2011). Circulating uncarboxylated matrix gla protein is associated with vitamin K nutritional status, but not coronary artery calcium, in older adults. J Nutr.

[27] Caluwe R, Vandecasteele S, Van VB, Vermeer C, De Vriese AS (2014). Vitamin K2 supplementation in haemodialysis patients: a randomized dose-finding study. Nephrol Dial Transplant.

[28] Shea MK, O’Donnell CJ, Hoffmann U, Dallal GE, Dawson-Hughes B, Ordovas JM, Price PA, Williamson MK, Booth SL (2009). Vitamin K supplementation and progression of coronary artery calcium in older men and women. Am J Clin Nutr.

[29] Dalmeijer GW, van der Schouw YT, Vermeer C, Magdeleyns EJ, Schurgers LJ, Beulens JW (2013). Circulating matrix Gla protein is associated with coronary artery calcification and vitamin K status in healthy women. J Nutr Biochem.

[30] Kidney Disease: Improving Global Outcomes (KDIGO) CKD-MBD Work Group (2009). KDIGO clinical practice guideline for the diagnosis, evaluation, prevention, and treatment of Chronic Kidney Disease-Mineral and Bone Disorder (CKD-MBD). Kidney Int Suppl.

[31] Kauppila LI, Polak JF, Cupples LA, Hannan MT, Kiel DP, Wilson PW (1997). New indices to classify location, severity and progression of calcific lesions in the abdominal aorta: a 25-year follow-up study. Atherosclerosis.

[32] Ueland T, Gullestad L, Dahl CP, Aukrust P, Aakhus S, Solberg OG, Vermeer C, Schurgers LJ (2010). Undercarboxylated matrix Gla protein is associated with indices of heart failure and mortality in symptomatic aortic stenosis. J Intern Med.

[33] Rennenberg RJ, Schurgers LJ, Vermeer C, Scholte JB, Houben AJ, de Leeuw PW, Kroon AA (2008). Renal handling of matrix Gla-protein in humans with moderate to severe hypertension. Hypertens Res.

[34] Krueger T, Schlieper G, Schurgers L, Cornelis T, Cozzolino M, Jacobi J, Jadoul M, Ketteler M, Rump LC, Stenvinkel P, Westenfeld R, Wiecek A, Reinartz S, Hilgers RD, Floege J (2014). Vitamin K1 to slow vascular calcification in haemodialysis patients (VitaVasK trial): a rationale and study protocol. Nephrol Dial Transplant.

[35] Cianciolo G, La Manna G, Donati G, Persici E, Dormi A, Cappuccilli ML, Corsini S, Fattori R, Russo V, Nastasi V, Coli L, Wratten M, Stefoni S (2010). Coronary calcifications in end-stage renal disease patients: a new link between osteoprotegerin, diabetes and body mass index?. Blood Purif.

[36] Cranenburg EC, Brandenburg VM, Vermeer C, Stenger M, Muhlenbruch G, Mahnken AH, Gladziwa U, Ketteler M, Schurgers LJ (2009). Uncarboxylated matrix Gla protein (ucMGP) is associated with coronary artery calcification in haemodialysis patients. Thromb Haemost.

[37] Hermans MM, Vermeer C, Kooman JP, Brandenburg V, Ketteler M, Gladziwa U, Rensma PL, Leunissen KM, Schurgers LJ (2007). Undercarboxylated matrix GLA protein levels are decreased in dialysis patients and related to parameters of calcium-phosphate metabolism and aortic augmentation index. Blood Purif.

[38] Jono S, Ikari Y, Vermeer C, Dissel P, Hasegawa K, Shioi A, Taniwaki H, Kizu A, Nishizawa Y, Saito S (2004). Matrix Gla protein is associated with coronary artery calcification as assessed by electron-beam computed tomography. Thromb Haemost.

[39] McCabe KM, Booth SL, Fu X, Shobeiri N, Pang JJ, Adams MA, Holden RM (2013). Dietary vitamin K and therapeutic warfarin alter the susceptibility to vascular calcification in experimental chronic kidney disease. Kidney Int.

[40] Brandenburg VM, Kramann R, Specht P, Ketteler M (2012). Calciphylaxis in CKD and beyond. Nephrol Dial Transplant.

[41] Chan KE, Lazarus JM, Thadhani R, Hakim RM (2009). Warfarin use associates with increased risk for stroke in hemodialysis patients with atrial fibrillation. J Am Soc Nephrol.

[42] Koos R, Mahnken AH, Muhlenbruch G, Brandenburg V, Pflueger B, Wildberger JE, Kuhl HP (2005). Relation of oral anticoagulation to cardiac valvular and coronary calcium assessed by multislice spiral computed tomography. Am J Cardiol.

[43] Price PA, Faus SA, Williamson MK (1998). Warfarin causes rapid calcification of the elastic lamellae in rat arteries and heart valves. Arterioscler Thromb Vasc Biol.

[44] Palaniswamy C, Sekhri A, Aronow WS, Kalra A, Peterson SJ (2011). Association of warfarin use with valvular and vascular calcification: a review. Clin Cardiol.

[45] Schurgers LJ, Joosen IA, Laufer EM, Chatrou ML, Herfs M, Winkens MH, Westenfeld R, Veulemans V, Krueger T, Shanahan CM, Jahnen-Dechent W, Biessen E, Narula J, Vermeer C, Hofstra L, Reutelingsperger CP (2012). Vitamin K-antagonists accelerate atherosclerotic calcification and induce a vulnerable plaque phenotype. PLoS One.

[46] Holden RM, Sanfilippo AS, Hopman WM, Zimmerman D, Garland JS, Morton AR (2007). Warfarin and aortic valve calcification in hemodialysis patients. J Nephrol.

[47] Krueger T, Westenfeld R, Ketteler M, Schurgers LJ, Floege J (2009). Vitamin K deficiency in CKD patients: a modifiable risk factor for vascular calcification?. Kidney Int.

[48] Spronk HM, Soute BA, Schurgers LJ, Thijssen HH, De Mey JG, Vermeer C (2003). Tissue-specific utilization of menaquinone-4 results in the prevention of arterial calcification in warfarin-treated rats. J Vasc Res.

[CR49] The pre-publication history for this paper can be accessed here: http://www.biomedcentral.com/1471-2369/15/145/prepub

